# Secure Authentication for Remote Patient Monitoring with Wireless Medical Sensor Networks †

**DOI:** 10.3390/s16040424

**Published:** 2016-03-24

**Authors:** Thaier Hayajneh, Bassam J Mohd, Muhammad Imran, Ghada Almashaqbeh, Athanasios V. Vasilakos

**Affiliations:** 1 School of Engineering and Computing Sciences, New York Institute of Technology, Old Westbury, NY 11568, USA; thayajne@nyit.edu; 2 Computer Engineering Department, Hashemite University, Zarqa 13133, Jordan; Bassam@hu.edu.jo; 3 College of Computer and Information Sciences, King Saud University, AlMuzahmiah 11451, Saudi Arabia; dr.m.imran@ieee.org; 4 Computer Science Department, Columbia University, New York, NY 10027, USA; ghada@cs.columbia.edu; 5 Department of Computer Science, Electrical and Space Engineering, Lulea University of Technology, Lulea 97187, Sweden; th.vasilakos@gmail.com

**Keywords:** remote patient monitoring, MSN, authentication, Rabin algorithm, FPGA implementation, security issues

## Abstract

There is broad consensus that remote health monitoring will benefit all stakeholders in the healthcare system and that it has the potential to save billions of dollars. Among the major concerns that are preventing the patients from widely adopting this technology are data privacy and security. Wireless Medical Sensor Networks (MSNs) are the building blocks for remote health monitoring systems. This paper helps to identify the most challenging security issues in the existing authentication protocols for remote patient monitoring and presents a lightweight public-key-based authentication protocol for MSNs. In MSNs, the nodes are classified into sensors that report measurements about the human body and actuators that receive commands from the medical staff and perform actions. Authenticating these commands is a critical security issue, as any alteration may lead to serious consequences. The proposed protocol is based on the Rabin authentication algorithm, which is modified in this paper to improve its signature signing process, making it suitable for delay-sensitive MSN applications. To prove the efficiency of the Rabin algorithm, we implemented the algorithm with different hardware settings using Tmote Sky motes and also programmed the algorithm on an FPGA to evaluate its design and performance. Furthermore, the proposed protocol is implemented and tested using the MIRACL (Multiprecision Integer and Rational Arithmetic C/C++) library. The results show that secure, direct, instant and authenticated commands can be delivered from the medical staff to the MSN nodes.

## 1. Introduction

Remote patient monitoring is becoming more common across the healthcare industry around the world and particularly in the United States with various medical conditions tracked when patients are away from the hospital. Wireless Medical Sensor Networks (MSNs) are cyber-physical systems (CPS) that have emerged as key building blocks to provide real-time and ubiquitous remote patient monitoring. This technology could potentially reduce the number of hospital admissions if adopted across the healthcare industry. It could also boost health outcomes, as certain medical issues could be addressed more quickly before a condition escalates. Hence, healthcare remote monitoring solutions could potentially lower medical costs across the country. Since communications between the medical staff and the devices that are monitoring the patient take place over the Internet, they are vulnerable to a variety of cyber attacks [1].

The excessive network resources, real-time response and smart monitoring with early notifications about the patients’ status are some of the requirements to be supported. The most effective and cost-efficient solution to achieve the aforementioned requirements is to deploy MSNs, also referred to as Wireless Body Area Networks (WBANs) [2,3]. Typically an MSN consists of sensors or actuators that are attached in, on or in the vicinity of a human body and that operate under the control of a master node that is often referred to as a smart device. The sensors report data to the medical staff on a periodic basis on a variety of body health indicators, such as blood pressure, temperature, heart rate, *etc*. The actuators execute therapeutic commands issued by the medical staff or an intelligent controller.

To overcome MSNs’ limitations (in computational power, data/energy storage and communication range/bandwidth) as standalone systems, MSNs have been integrated with cloud computing [4]. Moreover, storing and processing the reported data at local medical units limit its accessibility and complicates the system design [5]. Hence, integrating cloud computing with health-related systems comes to promote the gained performance by utilizing the abundant resources of data processing and storage offered by the cloud [6,7,8]. In fact, cloud computing-based mobile health monitoring is claimed to be 10-times more energy-efficient and almost 20-times faster than a standalone mobile health monitoring application [7].

Several challenges are facing this integration, including congestion, interference and coexistence issues, fast response, smart processing of the reported health-related data, supporting the maximum possible number of users, in addition to flexibility in operation and, most importantly, security [4,9,10,11]. In fact, data security is the largest obstacle that may impede the extensive usage of cloud-based MSNs. The researchers in [8] emphasized the importance of defining system-wide security mechanisms in human-centered systems to guarantee people’s privacy. Moreover, the researchers in [10,12] highlighted that security and privacy are amongst the most challenging issues for mobile cloud computing. It was also reported that the patient’s health could be seriously threatened by a malicious adversary [13], and using traditional security techniques may not be suitable, as it is recommended to offload the heavy security processing to the cloud or adopt lightweight cryptography [14,15].

**Figure 1:**
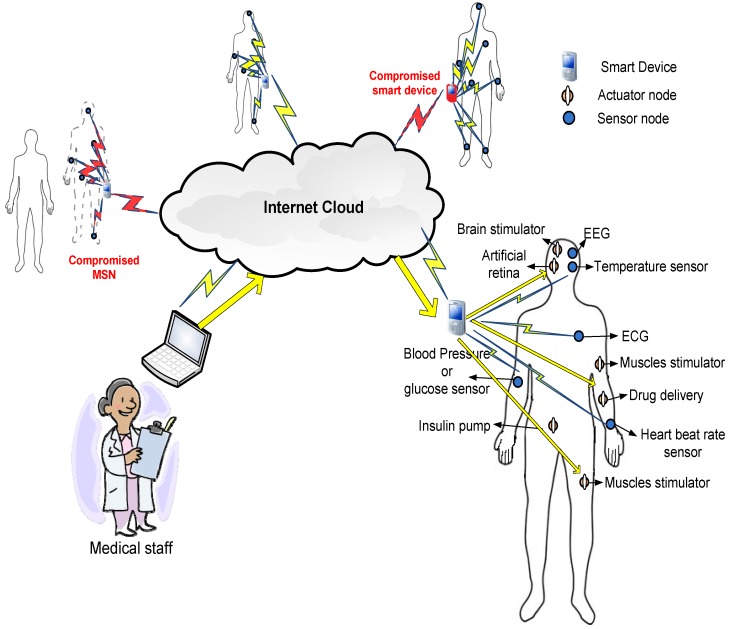
Remote patient monitoring through the MSN system.

Figure 1 shows the architecture of remote patient monitoring through the MSN system that we use to demonstrate the security protocol suggested in this paper. In Figure 1, each patient is represented as an MSN with tiny sensors or actuators reporting measurements or performing subtle actions. The MSN nodes are classified into two main types. The first type is sensor nodes that report data regularly to the smart device about the health vital signs, e.g., heart beat rate, temperature, blood pressure, *etc.* [2]. The second type is actuators that receive commands from the medical staff to perform actions and handle potential health problems, e.g., insulin pumps in the case of diabetes [16].

There are two main security concerns in MSN architectures. The first issue is to guarantee the authenticity and integrity of the commands issued by the medical staff to the MSN actuators. The commands involve actions that are performed by the MSN nodes and may have serious impacts on the human body. Hence, masquerading as a command or creating a fake one is considered a serious threat to human life. The second issue is to ensure the confidentiality of the reported data from the MSN sensors to the medical staff.

Researchers have argued that the security protocols proposed for traditional wireless sensor networks (WSNs) and mobile *ad hoc* networks (MANETs) are not directly suitable for MSNs [17]. MSNs have unique and challenging operational and security requirements, in particular being lightweight and having low delay. For instance, protocols that use complicated and computationally-expensive cryptography (e.g., elliptic curve [17,18,19,20]) are vulnerable to denial of service (DoS) attacks where the adversary can continuously flood the MSN nodes with fake commands that may exhaust their resources and preoccupy them with verifying fake commands.

**Figure 2:**
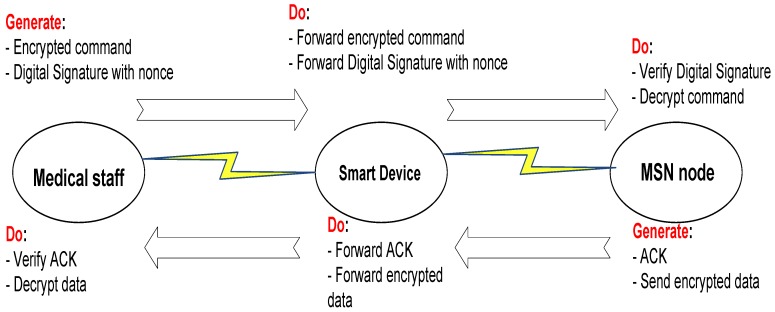
Security protocol details.

In this paper, we address the aforementioned security concerns using a lightweight public-key security protocol. This paper builds on and extends our work in Hayajneh *et al.* [1]. In particular, compared to [1], in this paper, we added a new Related Work Section in which we discuss and compare our work with other work in the literature. We have added a Threat Model Section in the paper that discusses the adversarial model, which takes into account the adversaries’ types and their power, in addition to the possible/plausible attacks that are performed by those adversaries. Our system incorporates three parties, namely the medical staff, the smart device and the MSN nodes (which implicitly represent the patient), and in this paper, we have added the details of the security protocol (Figure 2). The FPGA Implementation Section (FPGA stands for Field Programmable Gate Array) has been revised and expanded in this paper, where we added diagrams for the protocol design and the FPGA design flow, and also, we updated the results in Table 1. We compare our FPGA design with other designs in the literature. Moreover, we added an Experimental Testing Section in which we implement the protocol using the Multiprecision Integer and Rational Arithmetic C/C++(MIRACL) Cryptography library. We have also compared the Rabin to Rivest, Shamir, and Adleman ( RSA) public-key cryptosystem for different key sizes; the new results are summarized in Table 2. We have also expanded the Results and Comparisons Section and added a Security Analysis Section, which discusses the security properties of the proposed protocol.

The proposed protocol is based on the modified Rabin authentication algorithm, which has an extremely fast verification process compared to other public-key protocols. In fact, Rabin’s scheme was shown to be several hundreds of times faster and lighter than RSA [21,22]. The encryption that is performed by the MSN sensors is identical to the verification process. This implies that the MSN nodes are only required to perform the lightweight part of the Rabin algorithm. On the other hand, the heavy part of the Rabin scheme, *i.e.*, signature generation and data decryption, is performed by the medical staff or the smart device in some cases. In this paper, we modified the Rabin scheme to run components of the signature generation algorithm in parallel. This enhances Rabin scheme performance and makes it more suitable for MSN sensitive applications by reducing the potential response time.

Unlike the work in [23,24], in this paper, we do not focus on the user (medical staff in our case) authentication using a smart card or biometric authentication techniques. Moreover, most previous studies have assumed a well-behaved smart device that connects the MSN nodes via the Internet/cloud, a condition that might not be met in many cases. The researchers in [17,25,26,27,28] proposed efficient remote authentication protocols for MSNs. However, they assumed that the smart device is trustworthy and did not address the issues of having misbehaving smart devices or malicious MSN nodes. A smart device is usually a smart phone that is vulnerable to malware or a malicious application. The fact that G DATA Software (a specialist company in internet security and pioneer in the field of virus protection) security experts identified 440,267 new Android malware samples in the first quarter of 2015 [29] further supports our argument. Our authentication protocol addresses the issue of a compromised smart device efficiently by exchanging the signature between the medical staff and the MSN nodes, which is not shared with the smart device.

To evaluate the performance of the Rabin algorithm with the MSN, we implemented the algorithm with different hardware settings using the Tmote Sky mote. Moreover, the Rabin algorithm with and without the parallel settings is also implemented on an FPGA to evaluate its design and performance. Furthermore, the proposed protocol is implemented and tested using the MIRACL library. The aim is to prove that a lightweight public key can achieve the desired real-time response in cloud-based MSNs with high security and minimal power consumption.

The remainder of this paper is structured as follows. Section 2 discusses the related work in the literature. Section 3 presents the system and security modes, including the threat model. In Section 4, the Rabin algorithm is described. Section 5 illustrates the FPGA implementation where the experimental testing is presented in Section 6. Section 7 shows the testbed implementation. Section 8 presents the security analysis. Finally, Section 9 concludes the paper.

## 2. Related Work

In this section, an overview of the related work found in the literature regarding the security in MSN is presented. A light weight authentication scheme called TinyZKP is proposed in [30], which is based on zero proof knowledge. However, the proposed protocol targets sensor networks where the authentication protocol authenticates the sender sensor node. A scheme to capture data confidentiality in the cloud-assisted MSNs was proposed in [31]. Their goal was to achieve secure data communication between the cloud and MSNs without considering authentication and data integrity. He *et al.* proposed a lightweight trust management protocol for sensor networks in [32]. The proposed protocol was tested in a network of TelosB motes and was shown to improve the network performance and to protect it from malicious behaviors. A hybrid and secure priority-guaranteed MAC protocol for MSNs was proposed in [33]. The proposed protocol used a set of security keys to prevent unauthorized access to network resources.

The authors in [34] proposed a practical lightweight biometric approach to authenticate messages in MSN. They also developed an energy-efficient key agreement scheme that allows key sharing between MSN nodes with low overhead. Another light weight authentication protocol is found in [35]. The authors proposed a protocol that does not depend on prior trust among the nodes by exploiting physical layer characteristics unique to an MSN. Particularly, they utilized the distinct received signal strength (RSS) variation behaviors between an on-body communication channel and an off-body channel in the authentication process, which cannot be easily forged by attackers.

For key exchange and management in MSNs, Li *et al.* in [36] proposed a group device pairing (GDP) protocol, which is an authenticated key management protocol to construct the initial trust between the MSN devices and to distribute the secret keys between them. The work in [37] presented a Physiological Signal-based Key Agreement (PSKA) that allows neighboring nodes in an MSN to agree to a symmetric cryptographic key, in an authenticated manner, using physiological signals obtained from the human body.

The researchers in [25,26,27,28] proposed efficient remote authentication protocols for MSNs. However, they assumed that the smart device is trustworthy and did not address the issues of having a misbehaving smart device or malicious MSN nodes. Traditional public key cryptography algorithms are argued to be impractical in sensor networks because of the large computation and energy resources they require. In [38], they showed that it is possible to design public key encryption architectures with a power consumption of less that 20 *μ*W. They compared two architectures, the Rabin scheme and NTRUEncrypt. They showed that the Rabin scheme has no significant disadvantages compared to NTRUEncrypt.

In [39], the researchers showed that computing a 1024-bit RSA digital signature on an eight-bit sensor node requires on the order of 90 s and 10 s for signature verification. Moreover, [18] used signature-based Elliptic Curve Cryptography (ECC) on an eight-bit sensor node generating a 160-bit signature requiring on the order of 20 s, and 40 s are required for the verification. Moreover, In [40], the researchers proposed a symmetric key distribution and management scheme that is based on ECC for MSNs. They reported that ECC key agreement takes about 7.2 s on a Tmote Sky mote. According to the MSN requirements, this does not satisfy the delay constraints for MSNs and makes them vulnerable to DoS attacks [17]. The researchers in [19,20] reported similar results for elliptic curve Diffie–Hellman with a Tmote Sky mote.

A lightweight identity-based cryptography was presented by [41] to balance security and privacy with accessibility for MSNs. The researchers in [17] showed that [41] has several security weaknesses and efficiency problems, including node replication attacks, injection of false medical data and using the computationally-extensive ECC. The researchers in [42] proposed an ECC-based mutual authentication and access control protocol for MSNs. However, the study in [43] identified security flaws in [42] and showed that the scheme is susceptible to information leakage attacks. The work in [44] presented an efficient and adaptive mutual authentication framework for real heterogeneous WSN-based applications. It is also worth mentioning that MSNs are also vulnerable to attacks that cannot be prevented using cryptographic protocols, such as: jamming [45], packet dropping [46,47], wormholes [48,49] and localization [50,51].

In [17], they proposed a lightweight system to secure wireless medical sensor networks. The proposed system was built using symmetric cryptography and hash operation. Similar to other security protocols for MSN, [17] secure the communication between the user and the controller (smart device) and not all the way to the biosensor nodes. The work in [52] presented an efficient anonymous authentication scheme for wireless body area networks. The protocol was based on ECC and used identity-based authentication.

In [31], they proposed a scheme to capture data confidentiality in the cloud-assisted wireless body area networks. The goal is to achieve secure data communication between the cloud and MSNs. A secure patient-centric personal health information sharing and access control scheme in cloud computing is proposed in [53]. The proposed scheme was proven to resist various possible attacks and malicious behaviors. In [54], they proposed an MSN-cloud architecture to monitor a variety of biomedical conditions and to fulfill security goals for various medical services. In [55], they proposed a hybrid authentication and key establishment scheme for MSNs. The scheme uses symmetric cryptography in sensor/actuator modes.

In summary, our work is different from previous work that assumes a trustworthy smart device and only authenticates the commands between the medical staff and the smart device (e.g., [25,26,27,28]). Our protocol secures the commands all the way to the sensors, and a compromised smart device will not have the authority to forge or modify a command. Previous work that uses computationally-expensive cryptography (e.g., [19,20,40]) may exhaust the sensors limited resources and is vulnerable to DoS attacks. Our protocol is proven to be lightweight and is not vulnerable to these attacks. Moreover, protocols that rely only on symmetric cryptography will lack critical security properties, including non-repudiation, forward and backward secrecy, *etc*.

## 3. System and Security Models

**Figure 3:**
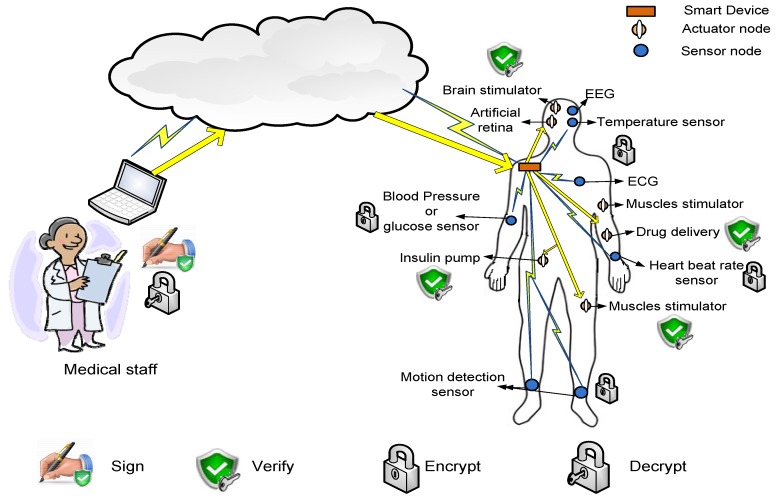
MSN security model.

In this section, the threat, system and security models are described. In particular, the main characteristics, relations, functionalities and the basic security aspects of the smart device, the MSN nodes and the transferred data are explored. The basic MSN security architecture used in this paper is depicted in Figure 3.

### 3.1. Threat Model

In this section, we introduce the threat model adopted for the proposed system. In particular, we discuss the adversarial model, which takes into account the adversaries’ types and their power, in addition to the possible/plausible attacks that are performed by those adversaries. As mentioned previously, our system incorporates three parties, namely the medical staff, the smart device and the MSN nodes (which implicitly represent the patient). These entities play different roles with respect to the threat model.

#### 3.1.1. Adversarial Model

The adversaries (or malicious parties) in our system are classified into two categories as follows:

*(1) Outsider Adversary:* This category refers to any adversary who is not part of the current active communication session. In other words, the adversary is not one of the three parties in our system (*i.e.*, medical staff, smart device, MSN nodes). We assume that all of the communications in the system take place over public channels. Thus, an outsider adversary can be a passive one who eavesdrops on the transmitted messages that are communicated between the various parties within the system or an active adversary who tries to modify the transmitted messages in a malicious way.

*(2) Insider Adversary:* This category includes the malicious parties within the system. As mentioned previously, the untrusted parties within the system include the smart device and the MSN nodes. However, the difference between them is related to the intended/unintended malicious behavior. Particularly, a compromised smart device is an untrusted intended party that performs any malicious behavior with the intent of harming the system. However, the MSN nodes could be both intended and unintended malicious parties in the sense that an adversary may replicate the sensor nodes and make them report invalid data to the medical staff. In this case, the MSN nodes are considered intended adversaries. On the other hand, consider the situation when the patient or the medical staff unintentionally transposes the MSN wearable sensors/actuators to a different patient from the one who is associated with these nodes in the system. In this case, the MSN nodes perform their job in an honest way all the time in terms of reporting the medical data and implementing the received medical commands; however, they are related to a different patient, and the patient profile is updated with incorrect data in the database. In this case, the MSN nodes are considered unintended adversaries.

Note that the medical staff is considered a trusted party and securely authenticated to the system; hence, all commands are being reviewed and all received information is being authenticated and checked carefully before being added to the patient profile. Moreover, in our threat model, we deal with probabilistic polynomial time (PPT) adversaries that are limited in their computational power and time.

#### 3.1.2. Potential Attacks

We consider the following attacks in our system:

- Impersonation: an adversary who tries to send fake commands or pretends to be the MSN nodes and sends invalid data to the medical staff to trigger them to issue urgent medical commands.
- Commands tampering: an adversary who modifies an issued command by the medical staff and tampers with its content.
- Commands replay attack: an adversary resends an old command over and over again to make the MSN nodes implement the same command multiple times.
- Patient’s privacy violation attack: an adversary who collects information about the current health status of the patient.
- DoS attack: an adversary who tries to disrupt system operation by sending fake commands that will consume the MSN node resources and disrupt them from performing legitimate commands.
- Operation delay attack: this attack is concerned with the real-time operation of the system, where an adversary holds the command/reported data for a while and then allows them to be sent to their ultimate destination; thus, a late valid command is not useful and affects the patient’s health in a negative way, especially in urgent situations.

Note that in all pf the above attacks, the intended adversary can be either an outsider adversary who listens to the communication channel and tries to carry out the attacks or a malicious smart device that is considered an intermediary between the MSN nodes and the medical staff and, thus, has the privilege of watching the entire traffic within the system. However, for the unintended adversary, we have the impersonation attack, where the MSN nodes are placed on a different patient from the one registered by the medical staff. Thus, they are reporting correct data about the body that does not match the correct patient profile.

### 3.2. System Model

In our system, we assume two types of smart devices: relay and smart master nodes. In the first type, the smart device collects the reported data from the MSN nodes, reports them to the medical staff, receives commands from the medical staff and sends them back to the intended MSN nodes. In the second type, the smart device has additional functionality to process the reported data from the MSN nodes and to generate suitable commands to handle a potential health problem faced by the patient. We do not recommend using the second type, as it assumes a trusted smart device, which is not a valid assumption, as elaborated earlier. In the first type, the smart device could be a regular MSN node, *i.e.*, it has limited hardware, software and power capabilities; while in the second one, it could be portable smart digital devices in which the computational and power resources are abundant.

The MSN nodes used in our system, as shown in Figure 3, are also classified into two types: sensors and actuators. The sensors are able to monitor the body health indicators and to generate data packets of the measured data. Examples of MSN sensors include sensors to measure the body temperature, the heart beat rate, the blood pressure, ECG, EEG, *etc.* [2,56]. On the other hand, the actuators are nodes that have the suitable hardware to perform actions specified by the commands sent by the medical staff at the cloud side or the smart device. Examples of MSN actuators are: artificial retina, insulin pump, automatic drug delivery, muscles stimulator, *etc.* [16]. Both MSN sensors and actuators complement each others’ work, *i.e.*, based on the reported data by the sensors, the needed action is performed by the actuators.

Moreover, as illustrated in Figure 3, the data transmitted through the network is also divided into two types: periodic reported data by the sensors and commands generated by the medical staff/smart devices. The periodic data are sent on a regular basis with different packet sizes based on the sensor type. For example, ECG sensors send large data packets and more frequently than temperature sensors [56]. For the commands, their frequency depends on the health status of the patients. Patients with critical situations may receive more commands compared to patients with stable body health signs.

### 3.3. Security Model

The details of the proposed security protocol are presented in Figure 2. The medical staff generates a command and nonce using a random number and time stamp. The command is encrypted using any symmetric key cipher, and a digital signature is generated for the command combined with the nonce. The smart device only forwards the command and the digital signature to the MSN node, which will verify the digital signature and use the nonce to verify the freshness of the command and detect a replay attack. As for encrypting the sensor-reported data, we suggest two options for this process. The first option is to use Rabin encryption; the sensors in this case will use the public key of the medical staff to encrypt the data with some secret concatenated with the data to prevent an attacker from sending fake data. The send option is to simple use symmetric cryptography with the same key that the medical staff used to encrypt the command. It is also worth noting that the Rabin encryption is identical to the signature verification, while Rabin decryption is identical to signature generation.

As elaborated earlier, one of the most critical security issues in the presented remote patient monitoring system with MSNs is to guarantee that the commands issued by the medical staff, usually located at the cloud side, to the actuators are not altered or fake. Due to the large number of MSN nodes, their wide distribution and the possibility for insertion/removal of MSN nodes, we argue that using public-key cryptography is the most efficient solution to achieve the desired security requirements. The MSN nodes need only to store the public key(s) of the medical staff that are authorized to issue controlling commands. Although being a less computationally-expensive option, using symmetric cryptography imposes new challenges in terms of key management and distribution. However, if a computationally-expensive public-key cryptographic system is used to provide all security services, then it will burden the MSN nodes and result in long delays and energy consumption.

Since the main security process that MSN nodes perform is to verify the authenticity of a signed message, we decided to use a public-key scheme that has a fast and efficient signature verification process. We found that the Rabin algorithm is an excellent candidate that satisfies our requirements [57]. The medical staff or smart device signs their issued commands using a digital signature with their private key. Then, the MSN nodes use the medical staff or smart device public key to verify the integrity and authenticity of the delivered commands.

Another important security service that can also be covered by our proposed security framework is the confidentiality of the reported data from the sensors to the medical staff. In this case, the MSN nodes encrypt their reported data using the medical staff or smart device public key. Fortunately, with the Rabin scheme, this process is identical to the verification process and is considered extremely light compared to the verification process of other public key algorithms.

A possible variation of the proposed protocol is to use the public key cryptography only to exchange authentication and encryption keys securely. The keys should be only exchanged between the medical staff and the sensors, which keep the smart device outside the loop. In this case, the protocol can be based on symmetric cryptography, which is lighter than asymmetric cryptography. It is critical in this case to generate and exchange new authentication and encryption keys each time the medical staff wants to communicate with sensors to ensure that the system satisfies the security properties that will be discussed in Section 8.

## 4. Rabin Algorithm

The Rabin algorithm was originally proposed by M. Rabin in [57], and sometimes, it is considered a special case of RSA. However, Rabin’s scheme was shown to be several hundreds of times faster and lighter than RSA [21,22]. This makes it an excellent candidate for our remote patient monitoring system with MSN.

### 4.1. Original Rabin

In this section, we present the details of the original Rabin public key signature scheme [58]. At first, each node should perform the following to generate a key pair:

1. Node A chooses two large random strong prime numbers, *p* and *q*.
2. Compute n=p.q.
3. A’s public key is *n*; the private key is $\left(p,q\right)$

Node A signs a message m∈M (where *M* is the message space) as follows:

1. Compute $\tilde{m}=R\left(m\right)$, where *R* is the redundancy function.
2. Compute $s=\sqrt{\tilde{m}}$mod*n*.
3. A’s signature for *m* is *s*.

Node B who receives *s* can verify the signature as follows:

1. Get A’s public key *n*.
2. Compute $\tilde{m}=s^{2}$mod*n*.
3. Verify that $\tilde{m}\in M_{R}$ (where $M_{R}$ is the image of *R*); if not, then reject the signature.
4. Recover $m=R^{-1}\left(\tilde{m}\right)$.

### 4.2. Modified Rabin

To overcome some of the issues with the Rabin scheme, a modified version of the Rabin signature is provided in [58].

At first, each node does the following to generate a key pair:

1. A selects two random primes p≡3(mod8) and q≡7(mod8) and computes n=pq.
2. A’s public key is *n*; the private key is d=(n-p-q+5)/8

Node A signs a message m∈M as follows:

1. Compute $\tilde{m}=R\left(m\right)=16m+6$.
2. Compute the Jacobi symbol $J=\left(\frac{\tilde{m}}{n}\right)$
3. If J=1, then compute $s={\tilde{m}}^{d}$mod*n*.
4. If J=-1, then compute $s={\left(\tilde{m}/2\right)}^{d}$mod*n*.
5. A’s signature for *m* is *s*.

For the Jacobi symbol $J=\left(\frac{\tilde{m}}{n}\right)$, we used the following recursive algorithm to compute it:

1. If $\overset{´}{m}=0$ and n=1, return J=1
2. If $\overset{´}{m}=0$ and n=0, return J=0
3. If $\overset{´}{m}=2$ and n≡1 or 7(mod 8), return J=1
4. If $\overset{´}{m}=2$ and n≡3 or 5(mod 8), return J=-1
5. If $\overset{´}{m}\geq n$, return $\left(\frac{\tilde{m}\%n}{n}\right)$
6. If $\overset{´}{m}\%2=0$, return $\left(\frac{2}{n}\right)*\left(\frac{\tilde{m}/2}{n}\right)$
7. If $\overset{´}{m}\%4=3$ and n%4=3, return $-1*\left(\frac{n}{\tilde{m}}\right)$
8. return $\left(\frac{n}{\tilde{m}}\right)$

Node B receives *s* and can verify the signature as follows:

1. Get A’s public key *n*.
2. Compute $\overset{´}{m}=s^{2}$mod*n*.
3. If $\overset{´}{m}\equiv 6$(mod 8), take $\tilde{m}=\overset{´}{m}$.
4. If $\overset{´}{m}\equiv 3$(mod 8), take $\tilde{m}=2\overset{´}{m}$.
5. If $\overset{´}{m}\equiv 7$(mod 8), take $\tilde{m}=n-\overset{´}{m}$.
6. If $\overset{´}{m}\equiv 2$(mod 8), take $\tilde{m}=2\left(n-\overset{´}{m}\right)$.
7. Verify that $\tilde{m}\in M_{R}$; if not, then reject the signature.
8. Recover $m=R^{-1}\left(\tilde{m}\right)=\left(\tilde{m}-6\right)/16$

Even with the Jacobi method, Rabin signature generation is not significantly more computationally intensive than RSA [58]. In this paper, we adopt the modified Rabin with the Jacobi algorithm and refer to it as Rabin. We further modify the signature generation of Rabin by running some of its components in parallel to improve its performance in terms of delay. The details of this modification are provided in the coming section. In our testing and evaluation, we only considered the signature verification and generation processes, as they are identical to the data encryption and decryption processes.

## 5. FPGA Implementation

The Rabin scheme was implemented on a Field Programmable Gate Array (FPGA) platform. Compared to other hardware platforms, FPGA implementation provides superior features, including speed, lower cost, faster development time, flexibility and configurability. Furthermore, the FPGA platform supports algorithm agility enabling switching algorithms during operations, the algorithm uploads and updates [59]. The Rabin scheme was implemented in Hardware Description Language (HDL), synthesized and downloaded on an Altera FPGA to demonstrate the correctness. The rest of the section explains the design flow, hardware design and results.

### 5.1. Design Flow

**Figure 4:**
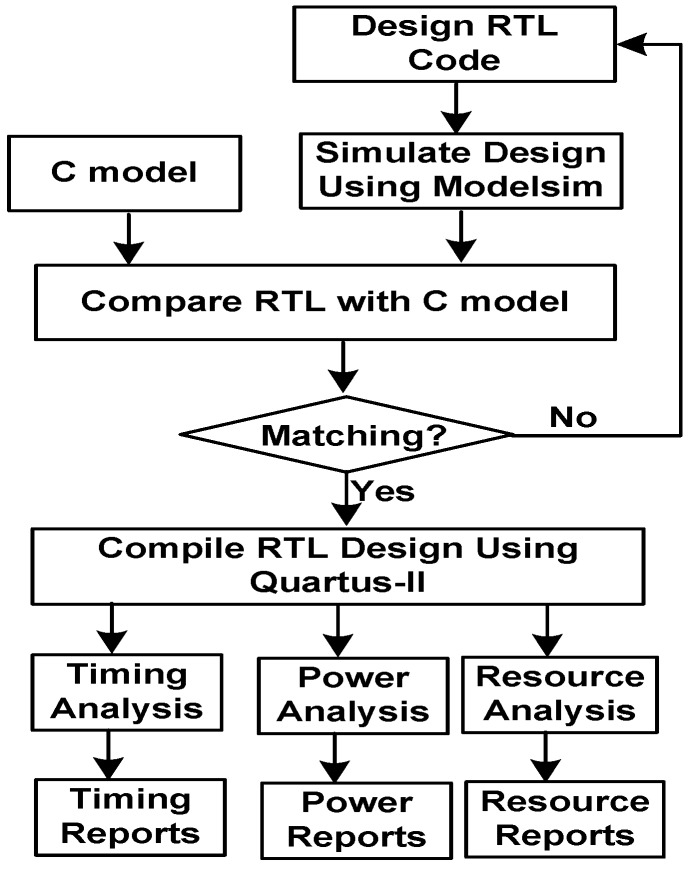
FPGA design flow.

The FPGA design adheres to a predefined design flow to illustrate and justify the design results. Published FPGA studies typically highlight their design flow [60,61]. Figure 4 illustrates the research design flow, which consists of the design and analysis phases. In the design phase, the Register-Transfer-Level (RTL) model is developed using the VerilogTM language. The RTL model is validated against the model that is programed in C programming language. The validation is performed by running RTL using Modelsim software [62] and then comparing the results of the RTL and C models. In the second phase, the design is compiled with FPGA software package Quartus-II [63] using the Cyclone-II FPGA device. The design timing is constrained to 50 MHz. Design inputs and outputs are constrained with 15 ns. The design is then synthesized and place-routed. Next, design timing, resources and power are analyzed. The timing analysis performs three types of maximum delays. The first type checks the timing delay from design inputs to flip-flops (*i.e.*, primary inputs to the register ($T_{su}$)). The second type checks timing delays from flip-flop outputs to design outputs (*i.e.*, register to the primary outputs ($T_{co}$)). The third type checks timing paths from flip-flop output to flip-flop input (*i.e.*, register to register (Clk-Clk)). The resource analysis step reports the number of resources required by the design. The resources are reported in terms of Logic Elements (LEs). An LE consists of: a four-input Look-Up Table (LUT) capable of implementing a four-variable function, a programmable register or both [63]. Power analysis computes the dynamic power dissipation, which includes clock power, register power and combinational logic power. The dynamic power calculation depends on the circuits’ activity [64]. The flow annotates the synthesized design with signal activity generated from Modelsim simulations. Modelsim captures the signal activity and saves it into a Value-Change Dump (VCD) file. The VCD file is then fed into Quartus-II to extract signal activities. The presented power analysis is similar to the power analysis used in other research works, such as [65,66,67,68,69].

### 5.2. Design

**Figure 5:**
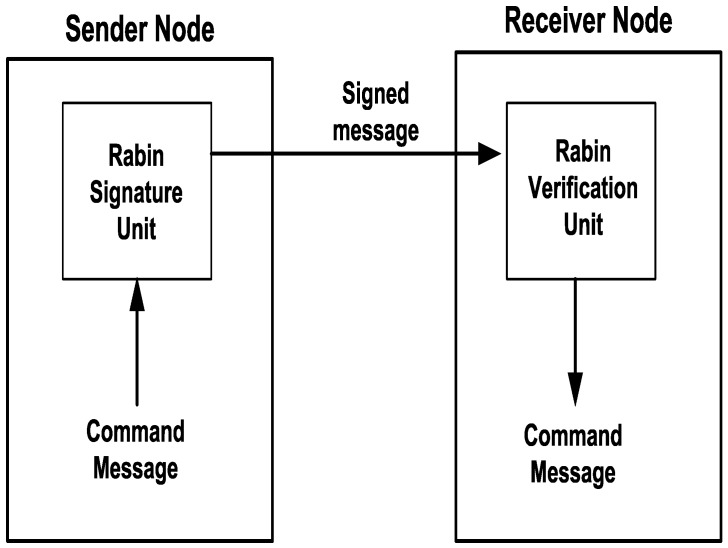
Design block diagram.

The FPGA design implemented signature and verification processes between the sender node and receiver node, as shown in Figure 5. The sender node employs the Rabin signature unit to encrypt the command message to generate a signed message. The receiver node employs the Rabin verification unit to extract the message command. Two signature units were implemented: the Rabin signature unit and the proposed parallel Rabin signature unit.

#### 5.2.1. Rabin Verification Unit

**Figure 6:**
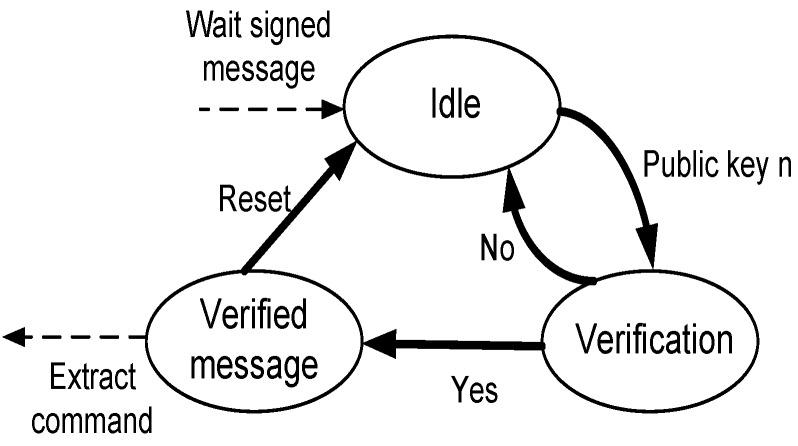
Rabin verification finite state machine (FSM).

The Rabin verification unit consists mainly of the finite state machine (FSM) illustrated in Figure 6. The FSM is initially at the idle state waiting for commands to arrive. After a message arrives, the receiver enters the verification state, obtains the public key *n* of the sender and runs the Rabin verification algorithm. If the message is verified, the required command is performed by the receiver node, and then, it returns back to the idle state. On the other hand, if the message is unverified, the receiver drops it and resets to the idle state.

#### 5.2.2. Rabin Signature Unit

**Figure 7:**
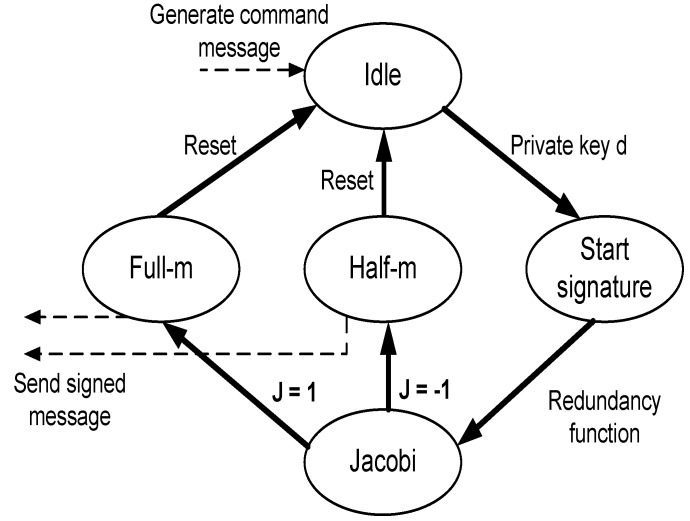
Rabin signature FSM.

Figure 7 shows the FSM of the Rabin signature design. Again, the sender’s FSM is initially at the idle state; when a certain action needs to be performed by the MSN nodes, a command message is generated. At this time, the sender moves to the next state to start the signature process using its private key. The result of the redundancy function is passed to the Jacobi state to compute the Jacobi symbol *J*. Based on the symbol value, the next state is determined; a value of one transfers the sender to the full-m state, while the value of −1 causes the transition to the half-m state. The two states differ in using either the *m* or m/2 value to generate the signature, as described earlier. After that, the sender resets and returns to the idle state, waiting for a new command to be signed and sent to the receiver.

#### 5.2.3. Parallel Rabin Signature Unit

**Figure 8:**
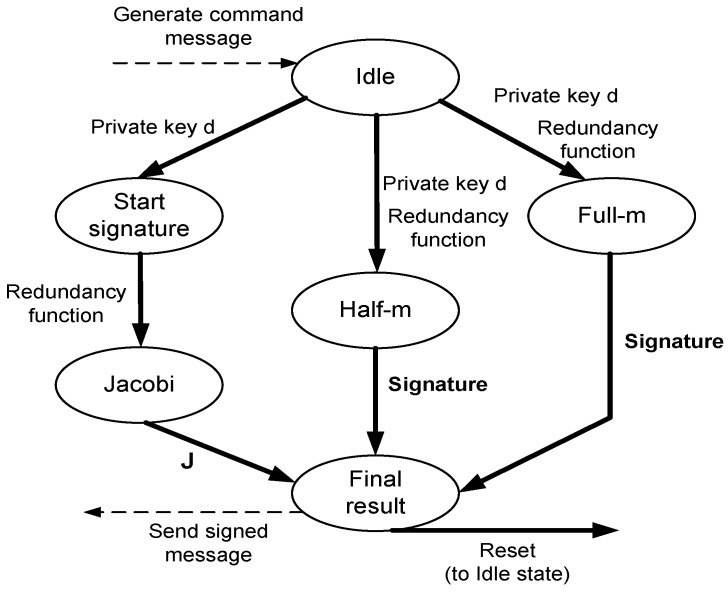
Parallel Rabin signature FSM.

The parallel Rabin signature consists of multiple blocks, as illustrated in Figure 8. The blocks, which are running at the same time, are: the Jacobi, full-m and half-m modules. As elaborated earlier, the idea is to make the signature generation process as fast as possible to decrease any potential delays, which is important in sensitive MSN applications. The Jacobi algorithm starts from idle state and ends up at the Jacobi state to compute the Jacobi symbol (Figure 7). By the time the Jacobi module is finished, both the half-m and full-m states are completed, and the one to be used for the signature value is selected (final decision) based on the Jacobi symbol value.

### 5.3. FPGA Results

In this section, we present RTL simulations and FPGA performance metrics. For the sake of clarity, we focus on basic performance metrics (*i.e.*, timing, area, power and energy), as a combination of metrics may cause confusion and can be sometimes misleading [14,70].

#### 5.3.1. Modelsim Results

**Figure 9:**
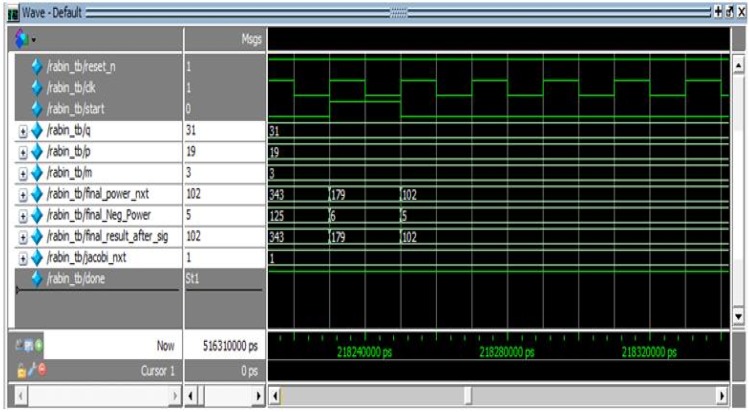
Modelsim wave diagram at the sender.

Modelsim simulations were performed to validate and verify the Verilog code of the sender (signer of the commands) and the receiver (verifier of the commands). Figure 9 shows the waveform for the input and the output signals generated by the sender for the parallel Rabin signature. Clearly, Figure 9 illustrates the correct behavior of the algorithm.

#### 5.3.2. Performance Results

In what follows, we discuss the FPGA implementation results in terms of hardware resources, timing, energy and power consumption.

Table 3 highlights the resource utilization results of the designs expressed in Logical Elements (LEs). The parallel Rabin understandably requires 64% extra LEs because of the added parallelism. Furthermore, the verification resources is less than both signature implementations, since it is a lighter and less complicated process.

The timing results for the different implemented designs are summarized in Table 4. The exhibited results include *T_su_*, *T_co_* and Clk-Clk. As shown, the computationally-laden signature algorithm is stretching the timing delays and lowering the design frequency to just above 2.6 MHz. On the other hand, the verification design does not experience lengthy timing delays.

Finally, the power and energy results are found in Table 1. The energy number was computed by multiplying the power by the timing per message. The timing per message was computed by multiplying the clock frequency (derived from the Clk-Clk timing in Table 4 by the number of cycles. Justifiably, the parallel Rabin consumes more power and energy than the standard Rabin. This is due to the fact that the added hardware and the increase in switching activity have contributed to the increase in power and energy; whereas the verification process in Rabin consumes less power and energy compared to the signature generation process. This justifies our selection for Rabin and requiring the MSN nodes to only perform the signature verification (or equivalently message encryption) process.

#### 5.3.3. Other Designs

Unfortunately, few articles reported the hardware implementation of the Rabin scheme. Murphy *et al.* [71] proposed a hardware-software co-design for the Rabin scheme using a Tyndall mote, which is a WSN prototyping platform with reconfigurable capabilities. The design was partitioned into hardware and software parts. The hardware was implemented using a Xilinix Spartan IIE FPGA. The software part was run on an eight-bit Atmel micro-controller. The study concluded that software implementation of the encryption is twice slower than hardware implementation. Unfortunately, no power or energy numbers were reported in the article.The work in [72] implemented ultra-power hardware for three public-key schemes: Rabin’s scheme, NTRUEncrypt and elliptic curve. The designs were implemented in TSMC 0.13*μ* CMOS standard cell technology. Algorithm parameters were selected to provide an equivalent level of security. The study concluded that Rabin is suitable for encryption and signature verification at the node. In terms of speed, Rabin-based encryption is the fastest, but Rabin-based decryption is the slowest, when compared to NTRUEncrypt and elliptic curve. As for the power and energy consumption, the Rabin design is the second best design. Future work should consider the modeling power and energy of the design based on parameters, such as the number of bits and packet size. The work in [73] modeled the energy of the lightweight block cipher using parameters such as block size and number of iterations.

## 6. Experimental Testing

The MIRACL Cryptography library [74] has been used in the experimental testing. It supports the needed building blocks of the designed protocol in addition to big number arithmetic, which are required in public key cryptographic-based protocols. In addition, this library is known to be significantly fast and is widely used to implement applications for embedded systems and low power devices, which makes it a good fit for MSN nodes. However, MIRACL does not include several protocols that are required in this paper, such as the Rabin, RSA and hashed message authentication code (HMAC) . Hence, we have coded these protocols and added them to MIRACL.

### 6.1. Protocol Implementation and Results

As for the implementation, the following parameter values were used: a modulus size of 1024 bits for the public key schemes and a medical command of size 40 bytes. The strong random generator (SRNG) in MIRACL has been used to generate random primes *p* and *q* randomly. This SRNG as described in the library documentation is proven to generate random numbers suitable for cryptographic applications. It needs both a strong seed and a random string of a size of 256 bytes to be initialized. For the seed, we have used the /dev/random utility in Linux to get truly randomly-generated seeds. For the random text, either the same utility can be used to get a highly random string or any random keystroke sequence from the keyboard can be read.

As for the key generation, for RSA, the public key exponent is selected first where different values have been tested to see the effect on the algorithm operation. Based on the selected value, then strong random primes are generated. For the Rabin algorithm, the strong random primes are generated while ensuring the basic keys properties, *i.e.*, p≡3(mod8) and q≡7(mod8).

For RSA, PKCS1.5 has been used to pad the sent messages where a 16-byte random number is added in front of the message. For the decryption, the Chinese remainder theorem has been used to speed up the decryption process. As for the hash function, it was implemented using SHA-3. Finally, the Rabin scheme for digital signature generation and verification has been implemented as described earlier in the paper.

After the system has been initialized and all needed keys are generated, the medical staff may generate a command that needs to be delivered to the MSN actuator. Both the command and the digital signatures are then generated and sent to the smart device, which will simply forward that to the MSN nodes, as shown in Figure 2. The protocol implementation has been debugged and checked for the result correctness of the generated keys, the encryption and decryption process and digital signature generation and verification.

All programs were tested on the same machine, and we are interested in the computational speed of the different protocol parts. We provide results for the process speed of the signature verification and generation. This is due to the fact that our target is to find a digital signature scheme that is fast to verify to support the real-time response of the system without consuming the nodes’ computational resources and power. The protocol has been tested using the following machine specifications: a laptop with core i7, 8 GB RAM and Ubuntu 12.04 operating system. The proposed protocol has been tested under different scenarios or key sizes: the first one denoted as Rabin uses this algorithm for the digital signature, and the other three modes all apply RSA with different key sizes: RSA-128 with a 128 key size, RSA-256 with a 256 key size, RSA-512 with a 512 key size and RSA-1024 with a 1024 key size.

The results found in Table 2 prove the objective of the proposed protocol, that is using the Rabin algorithm for the digital signature is much faster in the verification than RSA. As shown in the table, Rabin outperformed all RSA versions, including the low-power exponent RSA, where it achieves the least computational delay. Thus, the MSN nodes are able to verify the signature in a fast way, and so, the command will be executed faster to handle the patient’s health situation. In addition, the Rabin signature generation speed is comparable to RSA modes. However, this process is performed by the medical staff, which is not limited in resources, and it can be equipped with more powerful hardware components to speed up this operation; as opposed to the MSN actuators, which are limited in their resources and should have a small size to enable the construction of the MSN network. In addition, it is noted that as the key size in RSA is increased, the total delay of the medical staff operation is reduced. This is due to the fact that as the public key is larger, this means that the private key is smaller, and so, the signature generation process will be faster; this is verified by the results found in Table 2, which depict the speed of the signature generation and verification processes.

## 7. Testbed Implementation

In this section, we explore the testbed implementation used to evaluate the performance of the proposed security model. Similar to [75,76], we used TinyOS and the nesC language to develop the algorithms code and to upload them on Moteiv Tmote sky motes. In what follows, the main aspects of the tools used, the implementation details, the experiments’ setup and the obtained results are discussed.

### 7.1. Implementation Tools and Setup

TinyOS is an open-source operating system designed for wireless embedded sensor networks. It features a component-based architecture, which enables rapid innovation and implementation while minimizing the code size, as required by the severe memory constraints inherent in sensor networks. The TinyOS component library includes network protocols, distributed services, sensor drivers and data acquisition tools. nesC is an extension to the C language designed to embody the structuring concepts and execution model of TinyOS. To develop the code of the signature and verification algorithms, we translated the big number library from C to nesC. The translated library allows mathematical operations of numbers of a size of 512 bits.

Tmote sky is an ultra low power wireless module that can be used in rapid application prototyping. Tmote leverages industry standards like USB and IEEE 802.15.4 to interoperate seamlessly with other devices. It uses an 8-MHz Texas Instruments MSP430 microcontroller with 10 k RAM and 48 k flash. Although Tmote is one of the advanced customizable sensor nodes, its computational capabilities remain very limited, and this constraint must be considered while building the intended MSNs.

The packet format used in TinyOS is the same one used in 802.15.4. The default data field has a maximum size of 29 bytes. We created our own structure to use the data field of the packet as illustrated in Table 5.

The **src** field is the source address of the sending mote; pID is the ID of the current packet (used for fragmentation); Offset is the offset from the initial packet (used for fragmentation); message is the message to be sent; and finally, signature is the signature generated by the medical staff/smart device. As shown, the first three fields are of a size of one byte, while the message is of a size of four bytes, and the signature field has a size of 20 bytes. In our experiments, the commands signature is of a size of 64 byte; hence, we need four packets for each signature to be sent. To enable us to view the packets sent by the motes, we ran the Serial Forwarder on the ComPort of the mote and the Listener tool available in TinyOS.

### 7.2. Experiment Setup

**Figure 10:**
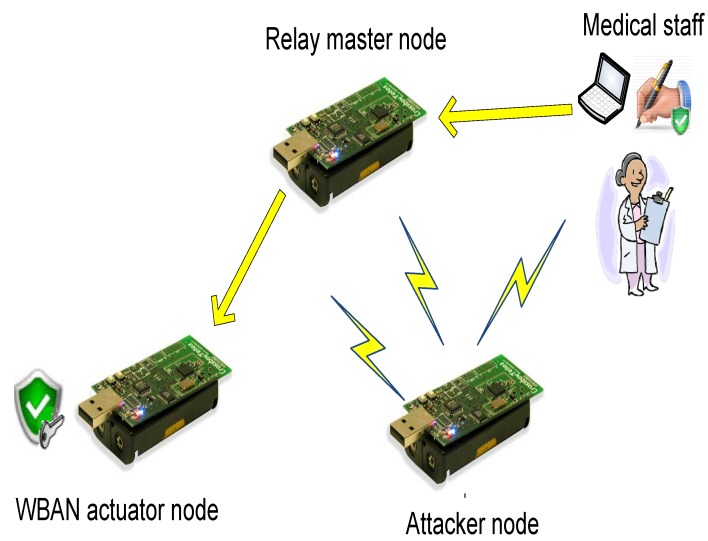
First testbed scenario.

**Figure 11:**
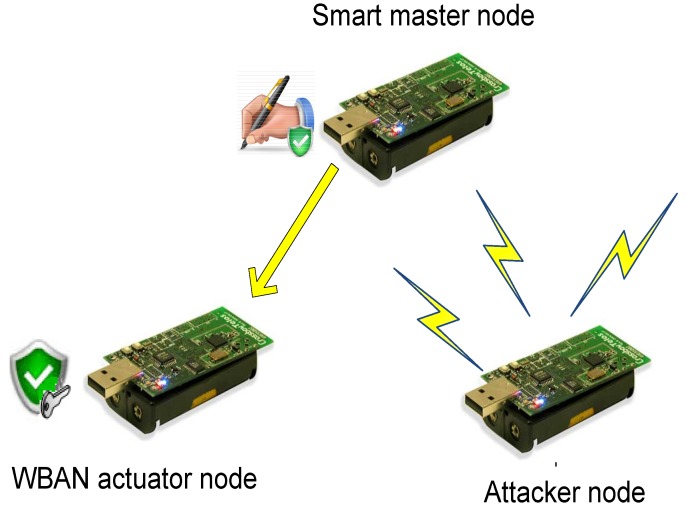
Second testbed scenario.

We built a testbed that contains three motes: the smart device node, the MSN node and an attacker node. The testbed has been configured to depict two different scenarios. In the first one, shown in Figure 10, the relay smart device is tested. However, in the second scenario, the same mote is configured to mimic the smart mote functionality that generates signed commands; see Figure 11.

The MSN node is the receiver mote that runs the verification algorithm. Two of the LEDs found on the Tmote sky are used to indicate whether the verification is successful or not as follows: the green LED turns ON if the signature is verified, and the red LED turns ON if the verification of the signature fails. The third blue LED can also be used to indicate that the node is sending or receiving data.

The relay smart device node is a mote running TOSBase, which is an application in TinyOS that makes the mote act as a bridge between the serial device (a PC) and the radio link (our wireless network). This application includes queues in both directions to guarantee that once a message enters the queue, it eventually leaves on the other interface. On the mote, this can be visually observed for every packet received and successfully bridged by the toggling of the green LED.

The smart device mote uses its own public/private key pair and runs the signing algorithm to generate the signature. We programmed the smart device mote to send the signature in four fragments, where one fragment is sent every 400 ms. The receiver mote collects these fragments in a global variable, putting each piece in its correct position. Once it receives all four fragments, it runs the verification algorithm and indicates the result on the LEDs, as mentioned before. Finally, we programmed the attack mote to impersonate the smart device and to send a bogus signature to the receiver mote.

### 7.3. Results and Comparisons

As mentioned earlier, we used big numbers of a size of 512 bits for all variables used by the signature and all intermediate calculation. Generally, public key authentication incorporates complex computations, which results in slow performance and high power consumption. Therefore, we evaluated the proposed security model in terms of the needed operations and time to sign/verify the commands.

In terms of computational requirements, the Rabin scheme has only one expensive operation on the receiver mote, which is squaring the received signature modulo the public key. Note that this operation is much less expensive than the two expensive operations that are performed during the generation of the signature, *i.e.*, computing the Jacobi symbol (which is a recursive function that uses modulo), and the modular exponentiation. However, this is a concern for the smart device node that is an MSN node (limited hardware resources). If the medical staff is performing the command signing process, the signing complexity is not an issue where high power devices are used for processing. However, in this case, achieving a rapid response with minimal delay is still desirable.

We note that the security of our algorithm relies on the problem of factoring a large integer. Similar to breaking the RSA, Rabin’s scheme is broken under a chosen-message attack. More specifically, if an attacker can choose two messages and obtain their signatures from our signing motes, the signature of the product of the two messages will be the product of the two signatures of the original messages.

The above attack could be harmful for sensor networks in two ways. First the attacker can use it to mount a denial of service attack on a certain mote. This is because using a valid message and signature pair, he or she can force the mote to compute the verification algorithm, therefore consuming its resources and causing a delay in processing other legitimate messages. On the other hand, construction of a message from the product of two messages could allow a prudent attacker to obtain a message that he or she can use to trick the receiver into believing the received message is reasonable. For example, in a fire monitoring application, which relies on temperature readings, an attacker can add the two temperatures, causing the receiver to trigger a false alarm.

For the second scenario with the smart device node, we obtained a signature generation time average of 22 s. The verification time on the other hand took less than 1 s. This result is very encouraging, as it reduces the computational requirements on the receiver to verify an incoming signature. It also reduces the effect of a DoS attack in the case of a malicious node sending bogus signatures or packets. On the other hand, using the parallel settings proposed in the previous section, the average signature generation time was reduced to 5 s.

We note that these signing and verification times are much lower than what can be achieved using other public key authentication protocols. In [39], the researchers showed that computing a 1024-bit RSA digital signature on and eight-bit sensor node requires on the order of 90 s and 10 s for signature verification. Moreover, [18] used signature-based Elliptic Curve Cryptography (ECC) on an eight-bit sensor node generating a 160-bit signature requiring on the order of 20 s and around 40 s for the verification. Table 6 compares the signature generation and verification timing for different schemes.

As for the energy consumption, in [38], they showed that it is possible to design public key encryption architectures with a power consumption of less that 20 *μ*W. They compared two architectures, the Rabin scheme and NTRUEncrypt, and the results showed that the Rabin scheme has no significant disadvantages compared to NTRUEncrypt. The latter is close to being practical on sensor nodes.

However, SNEP (Secure Network Encryption Protocol) and *μ* TESLA (Timed, Efficient, Streaming, Loss-tolerant Authentication Protocol) [77] used only symmetric key-based techniques to provide security. The main problem is that they require each node to be time synchronized with the base station and require key management functions and ample storage. This also causes a delay in the authentication process and might not be practical for real-time sensitive MSN applications. Further, the Merkle-Winternitz signature [78] used efficient one-time signature constructions that are computed quickly on sensor networks. Their problem is that they require high communication overhead on the order of 100–200 bytes per signature. Finally, in [79], they used a one-way hash function to conduct public key authentication in sensor networks. They assumed that they can exchange the one-way hash values of their public key securely prior to the deployment. Their results claimed an 86% energy savings compared to public key authentication.

Our public key approach differs from existing symmetric key approaches in that it only assumes the existence of public-key infrastructure (PKI) (or that the public/private keys are pre-installed authentically on the sensors). Moreover, there is no need for key establishment/management or re-keying.

Finally, the Rabin algorithm offers advantages compared to *μ* TESLA. *μ* TESLA is a broadcast authentication protocol, which is a lighter, efficient version of TESLA, and it is suitable for resource-constrained sensor networks [77]. Compared to *μ* TESLA, our scheme does not require time synchronization or any initialization messages to be sent. Moreover, *μ* TESLA requires the receiver to wait a predetermined amount of time, usually a few time slots, before starting the verification process of the received messages. In the Rabin scheme, receivers start verification immediately after receiving the commands, where the entire process takes around 1 s, making it suitable for real time applications.

## 8. Security Analysis

We analyzed the security protocol that was developed in this paper, and in what follows, we list the security properties and provide an informal proof or justification on how the protocol achieves these properties.

1. Command unforgeability: This security property implies that only the medical staff can create a valid medical command based on the patient’s current health status. In the proposed protocol, the medical staff uses a private key that no else knows. Hence, no one can forge a command.
2. Command non-reusability: An already issued/implemented command cannot be redirected to the MSN actuators to be implemented again. The medical staff will add a nonce, which is a combination of a timestamp and a random number, to each command. Hence, MSN nodes will detect any repeated or replayed command.
3. Confidentiality: Only the authorized parties are able to view the parts of the data that are sent to them. The commands are encrypted.
4. Mutual authentication: Both the medical staff and the MSN nodes are able to authenticate each other by proving their identities. The medical staff is authenticated to the sensors by using public-key cryptography, while the sensors are authenticated by sending and authenticated acknowledgment.
5. Integrity: All of the issued commands or reported medical data must be received correctly without being tampered with by any party. This is ensured by using the digital signature.
6. Availability: The existence of malicious parties in the system must not hinder the system operation and or affect the health status of the patient. A compromised master device cannot fake or modify a command.
7. Insider malicious actor detectability: The system is capable of detecting the existence of a malicious smart device or malicious MSN nodes and of responding accordingly. As elaborated earlier, the command is signed by the medical staff, and the signature is verified by the sensors.
8. Patients’ privacy protection: The system protects the privacy of the patient by preventing the disclosure of the reported data and the transmitted medical commands. All of the data are sent encrypted from the sensors to the medical staff and *vice versa*.
9. Forward secrecy: An adversary who has a subset of session keys cannot predict a subsequent session key. The protocol relied on public-key cryptography and, hence, provides forward secrecy.
10. Backward secrecy: An adversary who has a subset of session keys cannot predict a preceding session key. The protocol relied on public-key cryptography and, hence, provides backward secrecy.
11. Perfect forward secrecy: The compromise of the long-term key does not give the adversary any advantage of retrieving the previously-used session key and does not compromise the security of the old sessions.

We informally justified how the proposed protocol achieves the aforementioned security properties and is immune to the plausible attacks that can be carried out by any insider/outsider PPT adversary. The proposed protocol uses randomly-generated keys that are set up during the system initialization. Both the medical staff and the MSN nodes are trusted parties for this purpose, and they are holding the long-term keys. However, for the smart device, the medical staff generates and signs the command with a key that is not known to the smart device. Hence, it is infeasible for any attacker to correctly generate these signatures for any command. Moreover, even though the smart device sees the sent commands, the smart device cannot construct a valid signature.

As for the digital signature, hashing the encrypted command, which is also concatenated with a random nonce, eliminates the chance of generating the same signature for different commands. As a result, even if the medical staff generates the same command for the same patient multiple times, each command is treated as a new session and then is encrypted using a different key and is concatenated with a different nonce. The same is applied to the MAC, since we are using different session keys each time a MAC is generated. The inclusion of a nonce, that is a combination of a timestamp and random number, with each command will prevent replay attacks and provide message integrity. In Table 7, we describe how the proposed protocol can defend against the potential attacks that we described in the pre-defined threat model in Section 3.1.

## 9. Conclusions

Security and privacy issues were typically highlighted as a major obstacle hindering the growing of remote patient technology. Patients’ cited data privacy and security as their main concerns regarding this new technology, as security is a critical issue for MSNs. The medical staff usually sends important commands to MSN actuators to perform critical actions. The authenticity and integrity of these commands is the most critical security issue. This paper presented a lightweight public key authentication scheme for MSN systems. To prove its efficiency, the Rabin scheme was implemented with different hardware settings using a Tmote Sky mote. The Rabin scheme with and without the parallel settings was also implemented on an FPGA to evaluate its design and performance. The implementation results showed that secure, direct, instant and authenticated commands can be delivered from the medical staff located at the cloud side to the MSN nodes located in/on the human body. Moreover, the suggested parallel setting of the modified Rabin signature generation significantly reduced the delays (by almost 80%), which is a critical issue in MSN applications. The performance of Rabin was further verified by implementing and testing it using the MIRACL library.

## Figures and Tables

**Table 1:** Power and energy results.

Algorithm	Time Per Message (μS)	Power (mW)	Energy Per Message (nJ)
Rabin verification	0.033	31.800	1.0
Rabin signature	11.377	36.368	413.7
Parallel Rabin signature	7.998	122.923	983.1

**Table 2:** Computation speed results.

Mode	Signature Generation (ms)	Signature Verification (ms)
Rabin	16.686	0.186
RSA-32	17.214	0.422
RSA-128	17.370	1.110
RSA-256	17.552	2.100
RSA-512	17.512	3.670
RSA-1024	17.490	7.040

**Table 3:** Resource utilization. LE, Logical Elements.

		LE Type		
Algorithm	LEs	Combinational	Register	Both
Rabin verification	8025	7915	67	43
Rabin signature	12,053	11,580	46	427
Parallel Rabin signature	19,781	18,877	47	857

**Table 4:** Timing analysis (in ns).

Algorithm	$T_{su}$	$T_{co}$	Clk-Clk
Rabin verification	1.5	14.1	5
Rabin signature	10.5	297.5	372.4
Parallel Rabin signature	8.7	376.8	371.2

**Table 5:** Modified packet format.

1	1	1	4	20
src	pID	offset	message	signature

**Table 6:** Signature generation and verification time comparison. ECC, Elliptic Curve Cryptography.

Algorithm	Verification	Generation
Rabin	<1 s	22 s
RSA	10 s	90 s
ECC	40 s	20 s
Parallel Rabin	<1 s	5 s

**Table 7:** Adversary model analysis.

Potential Attacks	Protocol Defense
Impersonation	The protocol can prevent an adversary from impersonating a sensor and sending fake data by requiring the sensors to encrypt the data with a secret key
Commands tampering	The protocol uses digital signatures, which prevent this attack
Commands replay attack	The medical staff adds a nonce, which is a combination of a timestamp and random number, to each command, which prevents replay attacks
Patient’s privacy violation attack	This attack is prevented as the commands and the reported data are required to be encrypted with a secret key
DoS attack	The protocol relies on lightweight cryptography, which helps to prevent an attacker from sending a large number of fake commands to cause a DoS attack
Operation delay attack	The timing results show that the protocol provides a fast response and prevents delay attacks
